# Increase in mechanical load and pro-fibrotic stimulation leads to fibrotic and hypertrophic remodeling in porcine living myocardial slices

**DOI:** 10.1038/s41598-025-28222-z

**Published:** 2025-11-21

**Authors:** Marco Bentele, Sophie Linke, Susanne Neumüller, Andrea Korte, Anika Gietz, Annette Just, Angelika Stucki-Koch, Angelika Pfanne, Junqing Liu, Jiahao Zhao, Cornelia Schwennen, Christian Homann, Christian Visscher, Michael Pflaum, Bettina Wiegmann, Christian Bär, Natalie Weber, Jan Fiedler, Thomas Thum

**Affiliations:** 1 Institute of Molecular and Translational Therapeutic Strategies (IMTTS), Hannover Medical School, Hannover, Germany; 2 Fraunhofer Institute of Toxicology and Experimental Medicine (ITEM), Hannover, Germany; 3 Fraunhofer Cluster of Excellence Immune-Mediated Diseases (CIMD), Hannover, Germany; 4 Lower Saxony Center for Biomedical Engineering, Implant Research and Development (NIFE), Hannover, Germany; 5 Institute for Animal Nutrition, University of Veterinary Medicine Hannover, Foundation, Germany; 6 Center for Translational Regenerative Medicine, Hannover Medical School, Hannover, Germany; 7 Dean’s Office for Academic Career Development, nextGENERATION Medical Scientist Program, Hannover Medical School, Hannover, Germany

**Keywords:** *(up to 6)*: Cardiovascular diseases, Fibrosis, Hypertrophy, Living myocardial slices, Fibroblast activation, TGF-β stimulation, Cardiac hypertrophy, Cardiovascular diseases, Cardiology

## Abstract

Heart failure (HF) is a growing global health concern, driven by factors such as increased mechanical load of the heart muscle tissue. This pathogenic condition is the result of remodeling processes, partially mediated by the cytokine transforming growth factor β (TGF- β1), with progressive deposition of extracellular matrix within the myocardium that ultimately increases tissue stiffness. To elucidate early cellular and molecular processes underlying cardiac remodeling, translational experimental models are required. Living myocardial slices (LMS) represent a promising ex vivo model with preserved multicellularity, and physiology. In this study, we developed a disease model with hypertrophic and fibrotic features in porcine LMS. For this, LMS were prepared from porcine left ventricular tissue and cultured under mechanical overload with or without TGF-β1 stimulation. LMS cultured under overload showed reduced contractile performance, while adding TGF- β1 resulted in higher collagen deposition in the tissue, increase in secretion levels of pro-fibrotic miR-21 into the culture supernatant and increased expression of pro-fibrotic and hypertrophic marker genes. Our findings demonstrate early fibrotic changes in pig LMS induced by both mechanical and chemical stimulations. This ex vivo model could provide valuable insights into the early changes in heart failure pathogenesis and could be utilized as a screening platform to validate treatment strategies targeting cardiac remodeling.

**Supplementary Information:**

The online version contains supplementary material available at 10.1038/s41598-025-28222-z.

## Introduction

The aging population, along with the increasing prevalence of risk factors such as obesity and hypertension, especially in developed countries, contributes to the growing burden of cardiovascular diseases (CVD). As a result, CVD continue to pose a significant challenge to global public health and remain a leading cause of morbidity and mortality across diverse populations^1^. Despite significant advancements in medical interventions and preventive strategies, CVD accounts for a staggering number of deaths annually, with over 17 million people succumbing to its effects worldwide^2^. CVD encompass a broad spectrum of conditions affecting the heart and vasculature, ranging from hypertension to congenital heart defects and cardiomyopathies. Of particular concern is the rising incidence of heart failure (HF), a multifaceted condition representing a complex syndrome with diverse etiologies and clinical presentations, often characterized by cardiac remodeling resulting in an impaired cardiac function^3–5^.

Cardiac remodeling is particularly influenced by hypertrophy and fibrosis, which are closely interconnected and can mutually drive each other’s progression^6–8^. Hypertrophy represents an adaptive response to chronic cardiac overload, such as that caused by cardiac overload or myocardial infarction^9,10^. Although initially beneficial, sustained cardiac overload can lead to maladaptive remodeling of the heart, challenging the contractile machinery, and ultimately contribute to the development of heart failure^11–13^. In response to hypertrophic stimuli, hormones like atrial natriuretic peptide (ANP) and B-type natriuretic peptide (BNP) are released as part of the body’s attempt to counteract the effects of cardiac stress. These hormones help mitigate hypertrophic signaling pathways by promoting vasodilation and natriuresis, thereby reducing the strain on the heart^14,15^. Clinically, elevated levels of BNP or its precursor are commonly used as biomarkers to assess cardiac stress and can indicate heart failure progression. However, the protective effects of these peptides may diminish with chronic cardiac overload, facilitating the progression of pathological remodeling^16^.

A hallmark feature of this pathological remodeling is the progressive deposition of extracellular matrix within the myocardium, known as cardiac fibrosis^17^. Representing a dysregulated wound healing response, cardiac fibrosis is characterized by the excessive accumulation of extracellular matrix (ECM) proteins within the myocardium^18^. This process is driven by the activation of cardiac fibroblasts, leading to the stiffening of the myocardium and impaired contractile function^19^. In response to injury or chronic stress, cardiac fibroblasts undergo activation and differentiate into myofibroblasts, a process characterized by the upregulation of early fibrotic marker genes such as *ACTA2*. Encoding for smooth muscle alpha-actin, a key protein facilitating the contractile function of myofibroblasts, it enables these cells to adopt contractile properties^20,21^. Once differentiated, myofibroblasts express a multitude of pro-inflammatory and pro-fibrotic paracrine factors. In early stages of fibrotic changes these factors include glycoproteins like fibronectin, crucial for cell adhesion and the assembly of the extracellular matrix, or tenascin C, a glycoprotein involved in tissue repair and remodeling. Together, expression of these genes promotes the migration of myofibroblasts to the site of injury and the deposition of extracellular matrix components^22,23^. In later stages, myofibroblasts secrete high levels of collagen and other ECM proteins, resulting in a disproportionate increase in ECM quantity and significant alterations in ECM quality. Excessive collagen deposition, particularly types I and III, disrupts the structural balance of the myocardium, leading to increased tissue stiffness^24,25^. The altered ECM composition also creates a microenvironment that sustains fibroblast activation and perpetuates the fibrotic response^26^. A key component of this microenvironment is the cytokine transforming growth factor-beta 1 (TGF-β1), which plays a central role in promoting and maintaining fibrosis^27^. TGF-β1 is primarily secreted by activated fibroblasts, macrophages, and endothelial cells in response to cardiac injury or mechanical stress^28,29^. It drives the differentiation of fibroblasts into myofibroblasts, stimulates the synthesis of ECM proteins such as collagen and fibronectin, and suppresses ECM degradation by inhibiting matrix metalloproteinases. This sustained TGF-β1 signaling amplifies fibrotic progression, further compromising cardiac structure and function^30^.

Studying the molecular and cellular mechanisms underlying hypertrophic and fibrotic changes in the heart, and elucidating the complex processes of fibroblast activation, is crucial to find novel therapeutic targets and requires sophisticated experimental models^31,32^. Although in vivo models are regarded as the highest standard for scientific research, their complex nature often obscures the distinct contributions and interactions of individual factors. However, conventional in vitro models, such as monolayer cell cultures lack the multicellular architecture and physiological relevance of native cardiac tissue^33^. In contrast, three-dimensional (3D) tissue engineering approaches, such as engineered heart tissues (EHTs) and organoids, provide more physiologically relevant models by incorporating multiple cell types and extracellular matrix components. However, these models often contain cells with immature phenotypes and may not fully replicate the structural and functional complexity of the native heart^34–36^.

A model that overcomes these challenges and bridges in vivo and in vitro models are living myocardial slices (LMS). LMS are thin sections of left ventricular tissue that preserve the heart’s native structural integrity. Given adequate electrical stimulation and defined preload conditions, LMS maintain the physiological ECM composition and contain the full spectrum of cardiac cell types, such as cardiomyocytes, fibroblasts, and endothelial cells. They serve as a versatile, experimental model of intermediate complexity, providing a platform for investigating disease mechanisms, drug responses, and therapeutic interventions, while preserving the native cellular microenvironment for studying dynamic responses^37,38^. While LMS can be obtained from various species, including rodents, large mammals and humans, tissue from larger mammals generally exhibits longer viability under culturing conditions^39–42^. The protocol further allows for the production of multiple slices from a single heart, providing numerous experimental samples and thereby minimizing the need for animal usage in research. This system offers a unique opportunity to study fibroblast behavior in a physiologically relevant context, providing insights that are closer to in vivo conditions compared to traditional 2D and 3D models^43^.

In this study, we utilized porcine LMS to establish a disease model representing features of hypertrophic and fibrotic changes in cardiac tissue. We investigated the LMS response to both physiological and pathological preload, as well as TGF-β1 stimulation, and validated the resulting phenotype in contractile, structural, and molecular experiments. Herein, our results show early fibrotic changes, including reduced contractile performance and disruptions in both extracellular and intracellular structures of LMS in response to mechanical and chemical triggers. Thus, this model holds immense potential for advancing our understanding of early alterations in heart failure pathogenesis and identifying novel therapeutic targets in HF development in the future.

## Materials and methods

### Animal experiments

All animal experiments were reported and performed in accordance with ARRIVE guidelines and approved by the Institutional Animal Care and Research Advisory Committee and permitted by the responsible local authorities in Lower Saxony (LAVES, Oldenburg, Germany; 2022/293). The hearts used in this study were retrieved from healthy, adult female pigs (German Landrace, 3–4 month old, weighing 35–40 kg) provided by the group of Dr. B. Wiegmann from the department of biohybrid-lungs. The animals were euthanized in accordance with relevant guidelines and regulations of § 4 of the Animal Welfare Act (sacrifice notification 2019/247). Adult pigs received intramuscular injection of 1 mL per 10 kg weight of animal Zoletil 100 (Tiletamine 50 mg/ml + Zolazepam 50 mg/ml, Virbac) and 1 mL atropine per animal (atropin sulfate 0.5 mg/ml, B. Braun, #1940711) and then were sedated by intravenous administration of 20 mL Narcofol (propofol 10 mg/ml; CP-Pharma, #1210). Euthanasia was performed by overdose with 20 mL Release (pentobarbital sodium 300 mg/mL, WDT), also applied intravenously. After confirming death, the thoracic cavity was opened, and the heart was quickly excised and immediately immersed in ice-cold modified Tyrode’s solution (composed of 30 mM 2,3-butanedione monoxime (ThermoFisher, #15037500), 140 mM sodium chloride (Merck, #7647-14-5), 12 mM potassium chloride (Sigma-Aldrich, #P9541), 0.33 mM sodium dihydrogen phosphate monobasic (Merck, #13472-35-0), 10 mM anhydrous D(+)-Glucose (ThermoFisher, #410955000), 10 mM HEPES (Sigma-Aldrich, #H3375), 1 mM magnesium dichloride (Roth, #HN03.2), and 0.9 mM calcium dichloride (Roth, #5238.2), dissolved in water and adjusted to pH 7.4). The hearts were stored at 4 °C until further processing.

Additionally, hearts from 7-week-old growing male and female piglets (⌀11 kg body weight, fattening hybrids) were obtained from the Institute for Animal Nutrition at the University of Veterinary Medicine Hannover, Foundation approved by and performed in accordance with relevant guidelines and regulations of the Lower Saxony State Office for Consumer Protection under the approval number 33.19-42502-04-23-00448. Piglets were anesthetized with an intramuscular injection of ketamine (20 mg/kg, Ketamin 10%, Serumwerk Bernburg, #3160) and azaperone (4 mg/kg, Sedanol, Wirtschaftsgenossenschaft Deutscher Tierärzte, #25684) and subsequently euthanized via an intracardiac injection of T 61 (Embutramid 200 mg, Mebezonium iodide 50 mg, Tetracaine hydrochloride 5 mg; 4–6 mL/50 kg, T 61, MSD / Intervet Deutschland GmbH, #702020). Once respiration ceased and no heartbeat was detectable by auscultation, the major neck vessels were severed to exsanguinate the animals, the thoracic cavity was opened, and the hearts were excised. The hearts were promptly placed in sample containers filled with ice-cold, modified Tyrode solution and stored at 4 °C until further processing.

### Preparation of living myocardial slices (LMS)

The preparation and culture of myocardial slices was performed according to our previous work and Hamers et al. 2022 ^44,45^. In summary, modified Tyrode’s solution was cooled (4 °C) and used for tissue storage and processing. From the obtained pig heart, left ventricular tissue blocks were prepared, embedded into a 4% low-melting agarose (Sigma-Aldrich, #A9414), and glued onto the specimen holder with the epicardium faced down using surgical histoacryl glue (Braun GmbH, #1050052). Subsequently, the tissue blocks were fitted into a temperature-controlled tissue bath filled with 4 °C modified Tyrode’s solution, and sliced into 300 μm thick sections using high-precision vibrating microtomes (Model 7000SMZ-2, Campden Instruments LTD). For this, the vibratome was equipped with a ceramic blade (Campden Instruments LTD, #755-1-C) and operated with a vibration amplitude of 2 mm, a blade frequency of 80 Hz, and advance speed of 0.03 mm/s. Once the tissue cuts were acquired, the sections were trimmed into approximately 7 × 7 mm LMS using a razor blade according to the cardiac muscle fiber alignment. Lastly, plastic triangles were attached perpendicular to the cardiomyocyte alignment with surgical histoacryl glue, then transferred to MyoDish 1 Tissue Culture System (InVitroSys), also referred to as biomimetic cultivation chamber (BMCC).

Ex vivo culture of LMS at physiological preload and overload.

For LMS cultivation, pre-warmed and pre-equilibrated Medium 199 (Sigma-Aldrich, #M4530) supplemented with 1:1000 insulin-transferrin-selenium (ITS 100x liquid media supplement, Sigma-Aldrich, #I3146), 3% penicillin-streptomycin (Gibco, #15140), 20 µg/mL ascorbic acid (Sigma, #A1300000), 4 nM adrenaline (Sigma, #Y0000882), 4 nM noradrenaline (Sigma, #N1100000), 100 nM dexamethasone (Selleckchem, #S1322), and 2,15 nM triiodothyronine (Sigma, #T2877) was transferred into the BMCC. Following a brief adaptation period in the chambers, LMS were unloaded to determine the magnetic sensor baseline value and LMS preload was adjusted. For the physiological preload each LMS was gradually stretched until reaching the maximum force amplitude (maximum-minimum force value) kept at this state in the ex vivo culture. For the pathological preload each LMS was gradually stretched beyond its maximum force amplitude value until the force amplitude started to decrease again and kept at this state in the ex vivo culture. Additionally, a set of LMS in both conditions was cultured with 50 ng/mL TGF-ß1 (R&D Systems, #7754-BH-025). Throughout the experiment, LMS were cultivated under continuous electrical stimulation at 0.2 Hz, 50 mA / 3 ms, and agitation (60 rpm) at 37 °C and 5% CO_2_.

### Analysis of the contraction parameters

The BMCC enables continuous monitoring of LMS contraction, allowing the collection of data for absolute force measurement as well as contraction and relaxation kinetics. Contraction recordings were acquired continuously for 72 h ex vivo and analyzed at 24 h intervals. Using LabChart 98 (ADInstruments Ltd), ten contraction peaks were averaged, to assess the following parameters: force amplitude, time to peak 100% (TTP), time to 50% of relaxation (HRT), maximum relaxation velocity, and decay constant of relaxation (τ). Force amplitude refers to the maximum force compared to the baseline generated during a single cardiac muscle contraction. Time to peak refers to the time taken for the force amplitude to reach its peak value. Max slope represents the steepest rate of force increase during the contraction phase. Half relaxation time is the duration it takes for the force amplitude to decrease to 50% of its peak value during relaxation. Lastly, the decay constant of relaxation (τ) describes the rate at which force declines during relaxation, reflecting how quickly the muscle returns to its resting state.

### RNA extraction and real-time qPCR (RT-qPCR)

For miRNA isolated from culture supernatants:

For RNA isolation from supernatants, the miRNeasy Serum/Plasma Advanced Kit (Qiagen, #217204) was used and manufacturer’s instructions were followed. MiRNA quantification was performed by reverse-transcribing RNA with the miRNA TaqMan MicroRNA Reverse Transcription Kit (Applied Biosystems, #4366597). The resulting cDNA was diluted 1:3 with dH2O and used for qPCR with Absolute Blue qPCR Mix (Abgene, #AB-4136/B) and specific TaqMan probes (ThermoFisher, #4427975). Reactions were carried out on either a ViiA7 or QuantStudio 7 Flex system (both from Applied Biosystems).

For RNA isolated from LMS:

For RNA isolation of whole tissue, LMS were homogenized in QIAzol Lysis Reagent (Qiagen, #79306) for 40 s at 5500 rpm using the Precellys 24 homogenizer (Bertin Technologies) and stored at -80 °C for further processing. RNA extraction was then carried out using the precipitation technique. Reverse transcription of 500 ng RNA was carried out using the iScript Select cDNA Synthesis Kit (Bio-Rad, #170–8897) with oligo dT primers. The resulting cDNA was diluted 1:5 with dH2O before RT-qPCR analysis using iQ SYBR Green Supermix (Bio-Rad, #1708882). RNA quantification was performed using a mixture of forward and reverse primer pairs (10 µM, Eurofins). RT-qPCR runs were conducted on a QuantStudio 7 Flex system (Applied Biosystems).

The RT-qPCR data were analyzed using the ΔΔ-CT method and presented as fold change relative to the reference gene *GAPDH* for mRNA and *cel-miR-39* for miRNA. Primer sequences can be found in Table 1.

**Table 1:** RT-qPCR primer sequences. Ssc – sus scrofa. Hsa – homo sapiens.

Target	Forward primer sequence (5′→3′)	Reverse primer sequence (5′→3′)
*ssc_GAPDH*	CTCCCCGTTCGACAGACAG	TGAAGGGGTCATTGATGGC
*ssc_COL1A1*	CGCCATCAAAGTCTTCTGCA	ACTCGAACTGGAATCCGTCG
*ssc_COL3A1*	GGAAGCTATTGAAGGAGGATGC	ACAAACTGCACAACATTCTCCA
*ssc_MMP9*	CGAGATGACCGACAGAAAGC	TCCGAGTAGTTTTGGATCCAGT
*ssc_MMP2*	CAAGTTTCCCGGAGATGTCG	CTCATGGTCTCGATGGTGCT
*ssc_ACTA2*	CAGAGCGGAGAAGCTGAGTC	CTGGTGCTTCACAGGGTCAG
*ssc_FN1*	AGAAGAGGCACAAGATTCGGG	GTCATCCGTGGGTTGGCTTA
*ssc_TNC*	ATCAACGCAGCCACAGACTT	AGGCGGTAACGGTCAAACTT
*ssc_TGFB1*	TAAAAGTGGAGCAGCACGTG	GGCGAAAACCCTCTATAGCC
*ssc_NPPA*	CCCACAAGTACTAAGCGAGC	AGGGCAGATCTATCGGAAGC
*ssc_NPPB*	CACCTGTTGCTGCTAGGATG	CGGAGGACTTGGAAGATGCT
**Target**	**Mature miRNA sequence**	**Manufacturer**
hsa-miR-21	UAGCUUAUCAGACUGAUGUUGA	ThermoFisher (ID 000397)
cel-miR-39	UCACCGGGUGUAAAUCAGCUUG	ThermoFisher (ID 000200)

### Histology

Following cultivation, LMS were fixed with 4% PFA for 2–3 h at RT and then stored in 0.2 M phosphate buffered solution (240 mL solution A (27,6 g NaH_2_PO_4_ * H_2_0 / 137,99 g/mol in 1000mL dH_2_O) + 760 mL solution B (28,4 g Na2HPO4 * 2H_2_0 / 177,99 g/mol in 1000 mL followed by embedding in paraffin wax. Horizontal tissue sections, 5 μm thin, were produced using a microtome (Leica; RM2235). These sections were then affixed to specimen slides (epredia; #J1800AMNZ) and fixed again with 4% PFA for 10 min at RT. A portion of the fixed samples underwent washing followed by staining for 8 min with filtered DELAFIELD’s Hematoxylin solution (Chroma Waldeck, #2 C-161). After an 8-min rinse in tap water, they were stained for 3 min in 0.1% Eosin solution (Eosin G 0,5% Roth; #X883, 1:5 diluted in dH_2_O) supplemented with Acetic acid glacial (Roth; #3738). For fibrosis assessment, the other portion of the fixed samples was stained with Picrosirius Red (PSR). Following the same fixation process as described earlier, the tissue was rinsed and stained for 30 min with PSR stain solution (0.1 g Siriusred (Waldeck; 1 A-280) dissolved in 100 mL Picric acid 1,2% (Chroma; #3E-086)). Subsequently to a brief rinse with dH_2_O, the slices underwent dehydration in ascending alcohol dilutions with isopropanol (2-Propanol, Roth, #6752.4) and were mounted with Eukitt mounting medium for microscopy. Microscopy was conducted using a Keyence microscope model BZ-X810, and bright field images were captured at various magnifications (10x, 20x and 40x). Image analysis was performed using Fiji ImageJ 1.54f software^46^.

### Wheat germ agglutinin (WGA) staining

WGA staining was performed on the microtome-cut tissue sections. Tissue permeabilization was achieved using 0.1% Triton X-100 (Sigma Aldrich, #93443) for 10 min at RT, followed by two washes with PBS (Dulbecco’s Phosphate Buffered Saline; Gibco, #14190) (5 min each). Sections were then incubated in the dark for 40 min at RT with a staining solution containing Hoechst (1:1000 dilution, Thermo Fisher #H3570) and WGA (1:100 dilution, Thermo Fisher, # W11261). After three additional PBS washes (5 min each), sections were mounted for fluorescence microscopy. Images were acquired with Keyence microscope and quantified using Fiji ImageJ software. For every condition 70 to 100 cells were measured.

### Immunofluorescence staining

For immunofluorescence staining, microtome-cut tissue sections were deparaffinized, rehydrated, and subjected to antigen retrieval using pre-warmed Dako Target Retrieval Solution (Agilent, #S1699). Sections were microwaved at 700 W for 7 min and 300 W for another 7 min, then cooled on ice for 30 min. After rinsing in distilled water, slides were immersed in PBS-Tween (Roth, #9127.2; 0.05% Tween-20 in PBS) for 5 min at room temperature. Sections were blocked in 80–100 µL of blocking buffer (5% goat serum [Agilent, #X0907], 1% BSA [Serva, #11930.03], 0.3% Triton X-100 in PBS) in a humidified dark chamber for 45–60 min at RT. After a brief PBS-Tween wash, slides were incubated overnight at 4 °C with primary antibody anti-αSMA (Dako, #M0851; 1:50 in blocking buffer), avoiding air bubbles. The next day, slides were washed in PBS-Tween (2×), then incubated for 1 h at RT in the dark with donkey anti-mouse Alexa Fluor 488 (Invitrogen, #A21202; 1:500 in 1% BSA, 0.3% Triton X-100 in PBS). Sections were rinsed in PBS-Tween (2×) and PBS (2×, 5 and 10 min), followed by nuclear staining with Hoechst 33,342 (Invitrogen, #H3570; 1:2000 in PBS) for 15 min at RT in the dark. Final washes were done in PBS (2×, 5 and 10 min). Slides were gently dried, mounted using ProLong™ Gold Antifade Mountant (Thermo Fisher, #P36930), and coversliped. Images were acquired using a Keyence microscope and analyzed with Fiji/ImageJ. Nuclei were counted in entire images, and αSMA-positive cells were quantified as a fraction of cells per total cell number.

### Protein analysis using Western blot

For protein isolation from whole LMS, tissue was homogenized for 20 s at 5000 rpm using Precellys24 in a mixture of Pierce RIPA buffer (ThermoFisher, #89900) and cOmplete™, Mini, EDTA-free Protease-Inhibitor-Cocktail (Roche, #11836170001). For protein isolation from the culture medium, one volume of the sample was combined with four volumes of cold acetone (-20 °C, Roth, #9372.5). The mixture was vortexed and incubated at -80 °C overnight. Subsequently, the sample was centrifuged at 13,000 x g for 10 min. The supernatant was removed, and the pellet was air-dried at room temperature for 30 min. Finally, the pellet was resuspended in cell lysis buffer. For western blot analysis, the samples were loaded onto polyacrylamide gels to separate proteins via SDS-PAGE, followed by transfer to Immun-Blot PVDF Membranes (Biorad, #1620177). The membranes were blocked with a 5% milk solution for 1 h and then incubated with primary antibodies overnight at 4 °C. After washing, they were treated with HRP-conjugated secondary antibodies for 1 h at room temperature, followed by additional washes. Protein bands were visualized using enhanced chemiluminescence (ECL) reagent (Biorad, #1705061). Details of the antibodies used are provided in Table 2.

**Table 2:** Antibodies used for Western blot.

Antibody/compound	Manufacturer	Catalog	Origin	Dilution
Anti-Collagen Type I antibody	Sigma-Aldrich	234,167	Rabbit	1 : 500
Anti-Vinculin antibody	Sigma-Aldrich	V9131	Mouse	1 : 2500
Anti-rabbit, HRP-linked antibody	Cell Signaling	7074	goat	1 : 10.000
Anti-mouse, HRP-linked antibody	Cell Signaling	7076	horse	1 : 10.000
Anti-mouse Alexa Fluor 488	Invitrogen	A21202	donkey	1 : 500
Anti-αSMA	Dako	M0851	mouse	1 : 50
Hoechst 33,342	Invitrogen	H3570	-	1 : 1000
Wheat Germ Agglutinin (WGA)	Invitrogen	W11261	-	1 : 1000

### Supernatant analysis/ LDH measurement

For assessing levels of extracellular lactate dehydrogenase (LDH) in the culture medium, 200 µL of supernatant was analyzed at the department of clinical chemistry at the Hannover Medical School, using the Cobas 8000, Module c701 (Roche). The results were normalized to the volume of the corresponding LMS.

### Statistical analysis

Statistical analyses were conducted using GraphPad Prism (versions 8 or 9). Normality of the data distribution was assessed using the Shapiro–Wilk test in GraphPad Prism (v9.3.1), and all datasets were confirmed to follow a normal distribution, justifying the use of parametric statistical tests throughout the study. One-way ANOVA was used for comparisons involving three or more groups, while two-way ANOVA was employed for analyses with two distinct variables. Post-hoc tests, utilizing Tukey’s or Dunnett’s methods, were performed. Mean values with standard deviation (SD) are reported unless otherwise stated. Throughout all experiments, statistical significance was defined as a p-value of equal to or less than 0.05 (*: *p* ≤ 0.05, **: *p* ≤ 0.01, ***: *p* ≤ 0.001, ****: *p* ≤ 0.0001).

## Results

### Pathological preload impairs contractile function of porcine LMS

**Fig. 1:**
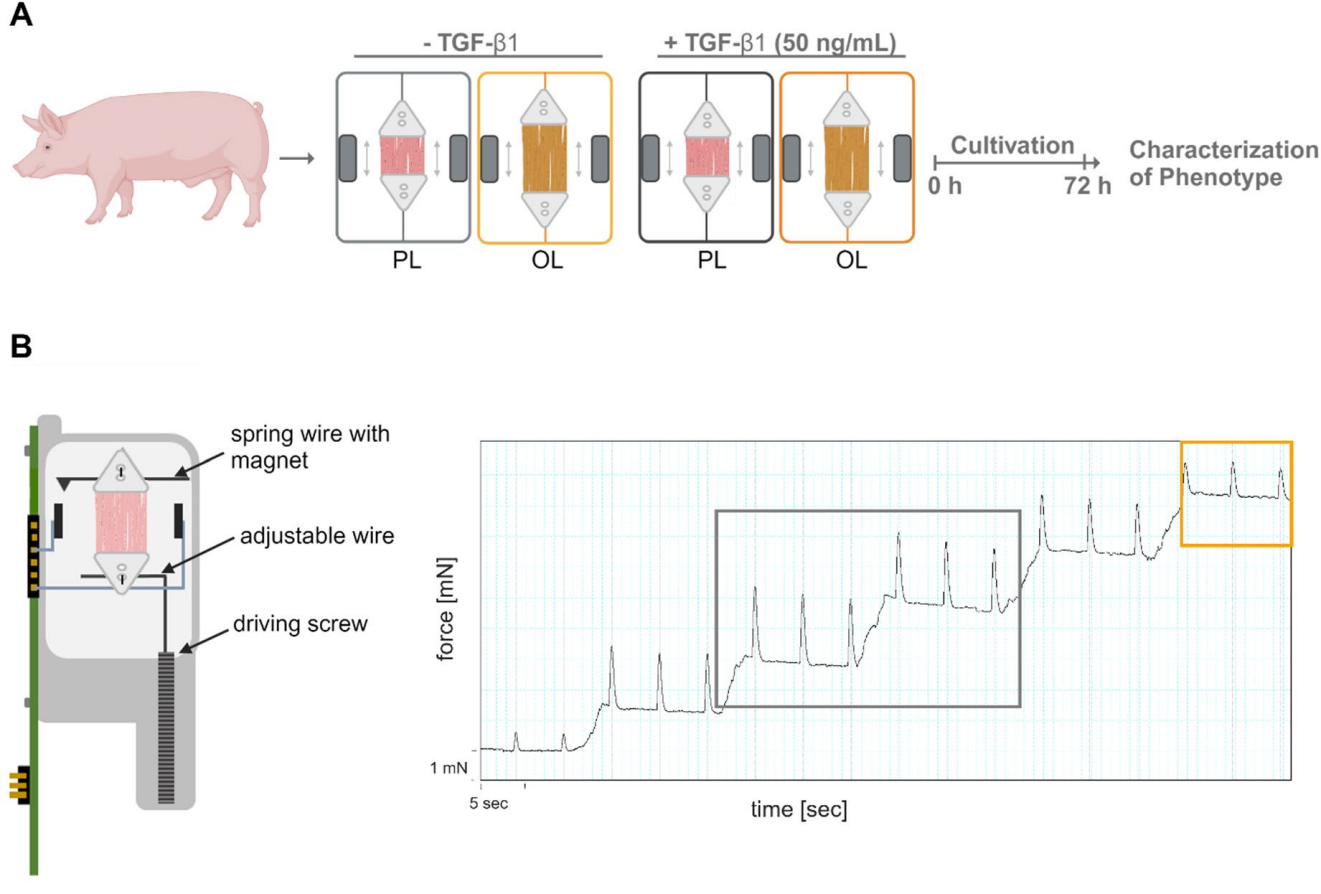
Establishment of a fibrotic and hypertrophic ex vivo model using porcine LMS. (**A**) Experimental set up. LMS were generated from porcine left ventricular tissue and cultured for 72 h, under either physiological load (PL) or overload (OL), with or without 50 ng/mL TGF-β1 added to the culture medium. (**B**) Left – Scheme of biomimetic cultivation chamber (BMCC) indicating the spring wire with magnet tip, adjusting wire and driving screw for adjustment of the preload. Right - Representation of preload adjustment in an individual LMS showing the increase of force development upon preload increase (grey box) with maximum force amplitude indicating PL condition, and decline of force amplitude after continuous preload increase, highlighting overload (orange box).

Heart failure (HF) is frequently caused by cardiomyocyte stress, which can be induced by factors such as pathological pressure overload in the adult heart^47,48^. In line, the cytokine TGF-β1 is a crucial factor for fibroblast activation and cardiac remodeling^30^. To replicate these conditions ex vivo, we used porcine living myocardial slices (LMS) subjected to either physiological preload (PL) or overload (OL), with or without TGF-β1 stimulation for 72 h, followed by assessment of myocardial function (Fig. 1A). LMS were maintained in biomimetic cultivation chambers that enable continuous recording of contractile force. This setup allowed us to define PL as the condition associated with maximal force generation and OL as the condition in which further increase in preload resulted in a decline in contractile amplitude (Fig. 1B).

The effect of the various culturing conditions on the contractile performance of the LMS was evaluated by analyzing the corresponding contractions recorded in the BMCC throughout the ex vivo culture. **Supplementary Fig. 1** shows a representative overview of all recorded contractions for individual LMS over 72 h, following preload adjustment. LMS cultured under PL exhibit a steady increase in force amplitude during the first 36 h of culture, with this force development remaining stable until the end of the experiment. In contrast, LMS cultured under OL reach their peak force amplitude between 24 and 36 h, after which the force declines steadily until the experiment concludes.

**Fig. 2:**
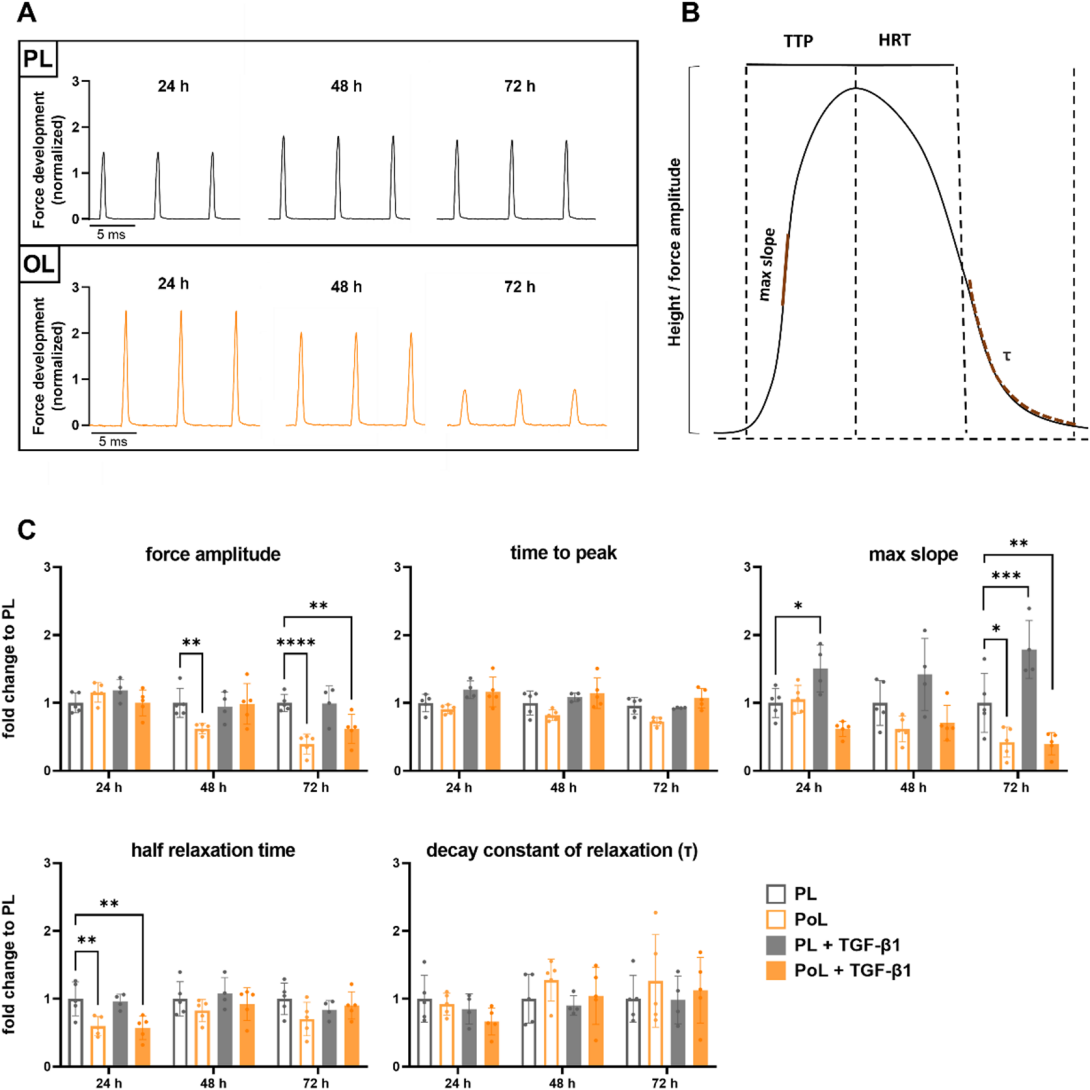
Overload reduces contractile function of porcine LMS. (**A**) Representative contractions from individual pig LMS cultured in physiological load (PL) and overload (OL) at selected timepoints 24 h, 48 h and 72 h. (**B**) Representative force transient indicating analyzed parameters: force amplitude, time to peak (TTP), max slope, half relaxation time (HRT) and decay constant of relaxation tau (τ). (**C**) Contractile parameters at defined time points obtained from force measurement of LMS in the BMCC. Each dot represents mean values of technical replicates (3–4 LMS) for one pig. *N* = 5 pigs, except LMS cultivated in PL + 50 ng/mL TGF-β1, here, *N* = 4 pigs (mean ± SD, two-way ANOVA; *, **, ***, **** = p value < 0.05, 0.01, 0.001, and 0.0001; ns = p value > 0.05).

To assess the functionality of the LMS in detail, we analyzed individual contractions at different time points after preload adjustment 24 h, 48 h, and 72 h (Fig. 2A). We analyzed several contractile parameters, as shown in Fig. 2B, namely force amplitude, time to peak (TTP), half relaxation time (HRT), max slope, and the decay constant of relaxation (τ). Figure 2C shows that the force amplitude of LMS cultured in OL decreases significantly after 48 h, and even more pronounced after 72 h, when compared to the LMS cultured under PL conditions. Moreover, overloaded LMS show a decreased max slope after 72 h compared to LMS under PL. Lastly, the HRT in LMS cultivated under OL is significantly decreased after 24 h, which is not significant anymore at the 48 h and 72 h time points. These effects are unaffected by the presence of TGF-β1. In fact, LMS incubated under PL and with TGF-β1 do not exhibit any significant impairment in contractile parameters with regard to force amplitude, TTP, HRT, or τ. Only an increase in max slope, significant after 24 h and 72 h, could be detected.

Taken together, these findings suggest that LMS cultured in OL show a significant reduction in contractile performance within 72 h, indicating an early onset of functional impairment resembling that observed in patients with progressive hypertrophic remodeling^49,50^.

### Overload and TGF-β1 stimulation replicate key characteristics of cardiac remodeling

**Fig. 3:**
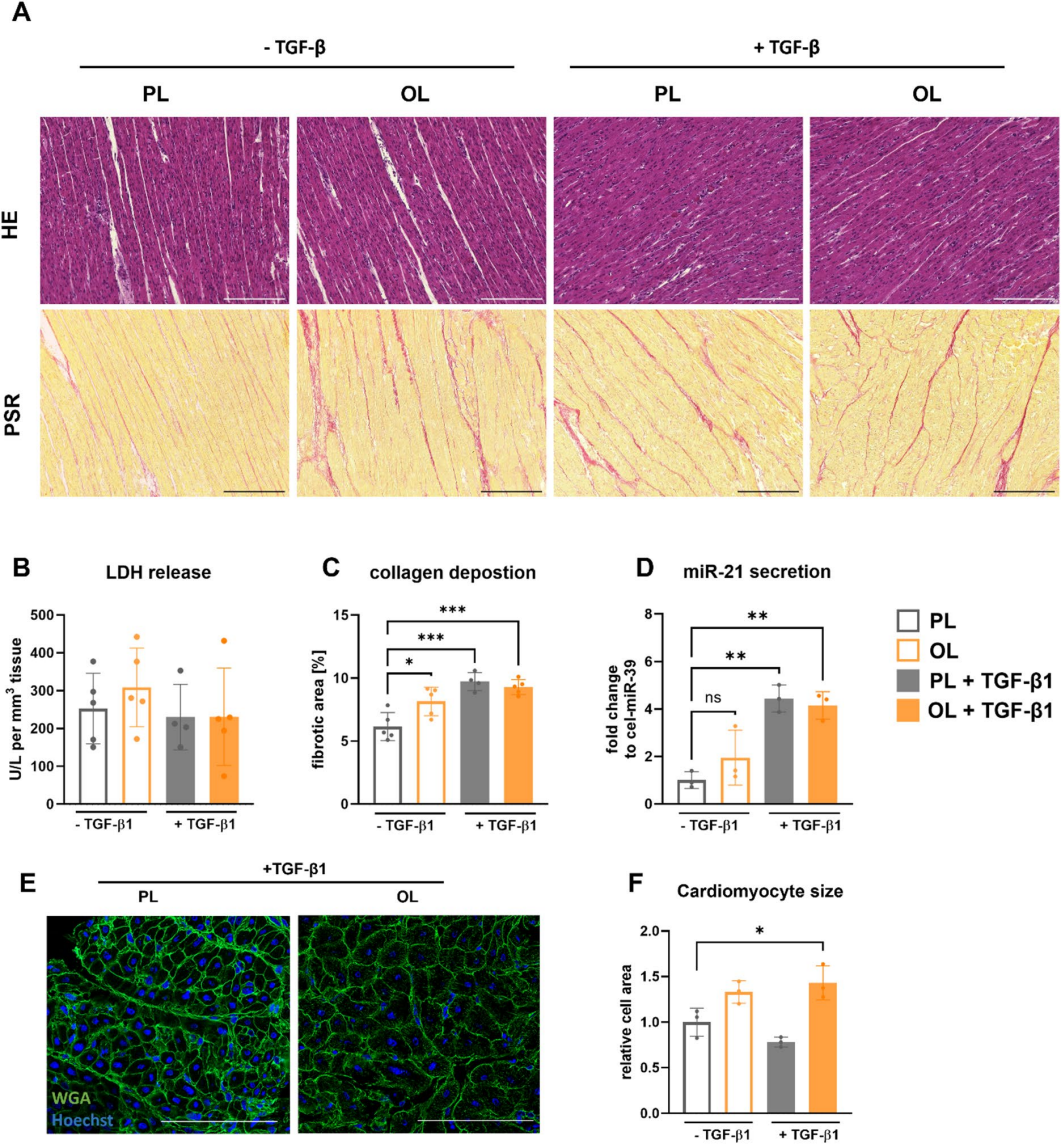
Overload and TGF-β1 stimulation of porcine LMS results in increased collagen I deposition and miR-21 secretion. (**A**) Representative images of hematoxylin and eosin (HE) and picrosirius red (PSR) staining. Scale bar = 100 μm. (**B**) LDH release measured from medium supernatant of cultured LMS. (**C**) Quantification of fibrotic area from PSR stainings. Each dot represents mean values of technical replicates (3–4 LMS) for one pig. *N* = 5 pigs, except LMS cultivated in physiological load + 50 ng/mL TGF-β1, here, *N* = 4 pigs. (**D**) RT-qPCR quantification of miR-21 secreted to the supernatant of culture medium, expression is normalized to the house keeper cel-miR-39. Each dot represents mean values of technical replicates (3–4 LMS) for one pig. *N* = 3 pigs. (**E**) Representative images of Wheat Germ Agglutinin (WGA) and Hoechst 33,342 staining from TGF-β1 treated, porcine LMS. Scale bar = 100 μm. (**F**) Relative cell area of measured cardiomyocytes. *N* = 3 pigs and 100 cells per condition. Mean ± SD, two-way ANOVA; *, **, ***, **** = p value < 0.05, 0.01, 0.001, and 0.0001; ns = p value > 0.05.

To assess the ability of our model to simulate the remodeling process observed in vivo following overload and TGF-β1 stimulation, we analyzed extracellular changes first, with regard to morphology, viability, and paracrine signaling. Exploring the tissue morphology and extracellular composition, we performed hematoxylin and eosin (HE) staining as well as picrosirius red (PSR) staining (Fig. 3A). The HE staining did not reveal any abnormalities in cell morphology and integrity. Additionally, measurements of lactate dehydrogenase (LDH) levels in the culture medium showed no significant increase in LDH release from LMS cultured under OL conditions or stimulated with TGF-β1 (Fig. 3B). Together, these results indicate that the tissue structure remains intact, with neither OL nor TGF-β1 stimulation causing significant tissue damage resulting in LDH release. However, after quantifying the fibrotic area in PSR stainings of the entire LMS (**Supplementary Fig. 2**), we observed an increased collagen deposition in LMS cultured under OL compared to those cultured under PL conditions (Fig. 3C). Additionally, TGF-β1 stimulation led to higher collagen deposition compared to the unstimulated samples. Specifically, LMS cultured with TGF-β1 showed larger collagen deposition, regardless of the preload condition, compared to LMS without TGF-β1 in the culture medium. Additionally, levels of microRNA-21 (miR-21) secreted into the culture medium were measured as an marker for pro-fibrotic changes indicating pro-fibrotic enviroment^51,52^. We could observe increased levels of miR-21 in LMS cultivated with TGF-β1, unaffected by preload condition **(**Fig. 3D**)**. Interestingly, although not statistically significant, LMS cultured under OL conditions without TGF-β1 exhibit a tendency towards increased miR-21 secretion. Lastly, Wheat Germ Agglutinin (WGA) stainings further showed slightly increased cardiomyocyte cross-sections in LMS cultured under OL and significantly increased cardiomyocyte cross-sections in LMS cultured with additional TGF-β1 stimulation, indicating early hypertrophic adaptations (Fig. 3E **and F**).

Overall, our results demonstrate that LMS cultured with TGF-β1 and OL, while maintaining tissue integrity and viability, possess key characteristics of cardiac remodeling, with regard to collagen deposition. Furthermore, the increased secretion of miR-21 into the culture medium in LMS cultivated with TGF-β1 highlights pro-fibrotic changes in the paracrine environment of the tissue.

### Overload and TGF-β1 stimulation induce expression of early fibrotic marker gene profile

**Fig. 4:**
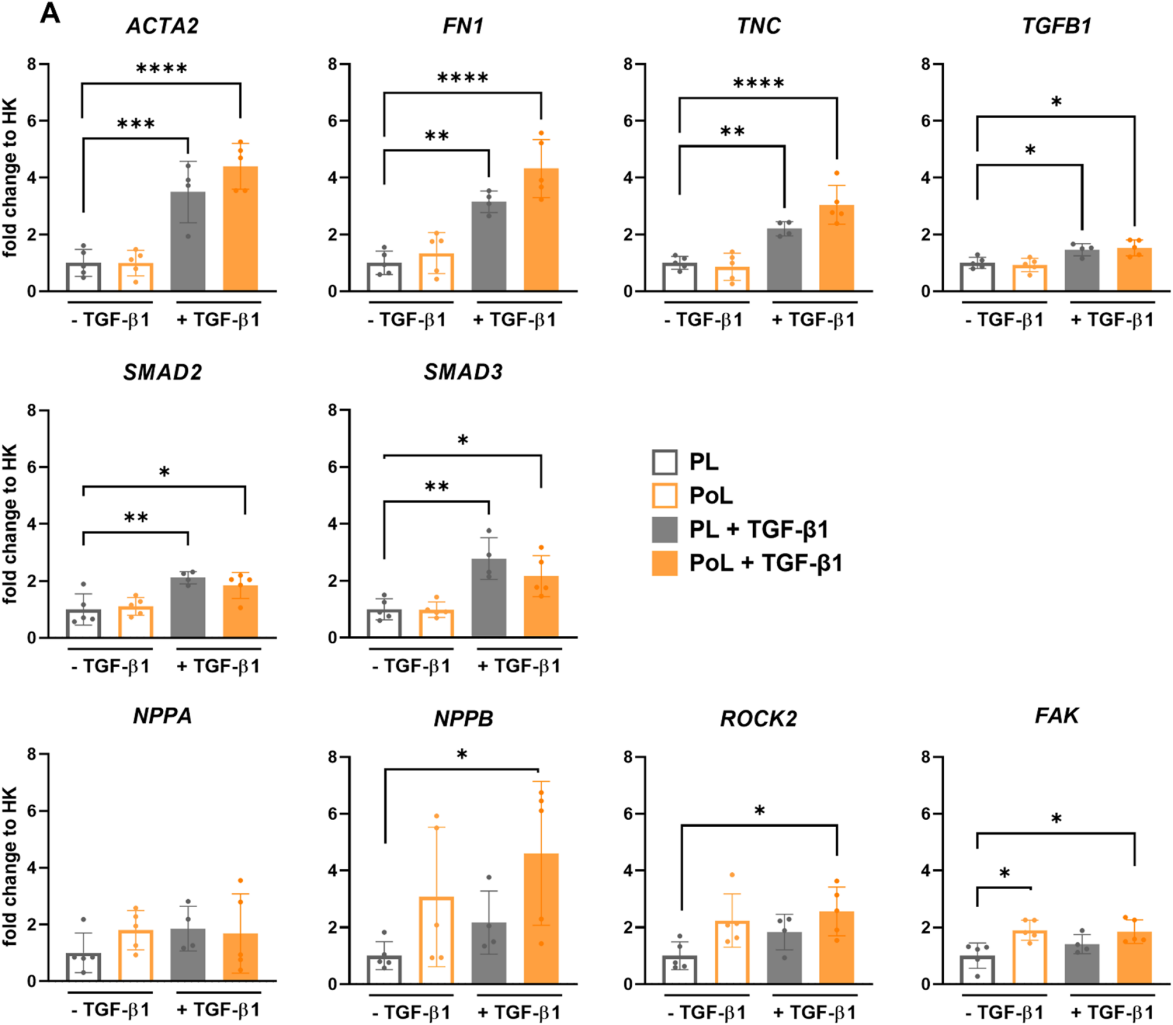
TGF-β1 stimulation results in increased expression of early, pro-fibrotic genes in porcine LMS. (**A**) RT-qPCR quantification of gene expression normalized to the house keeper (HK) gene GAPDH. Each dot represents mean values of technical replicates (3–4 LMS) for one pig. *N* = 5 pigs, except LMS cultivated in physiological load + 50 ng/mL TGF-β1, here, *N* = 4 pigs (mean ± SD, two-way ANOVA; *, **, ***, **** = p value < 0.05, 0.01, 0.001, and 0.0001; ns = p value > 0.05).

Considering the secretion of miR-21 into the culture medium and its known role in activating cardiac fibroblasts^51^, we aimed to examine the expression of multiple genes associated with fibroblast activation and early fibrotic changes in the ECM, indicating cardiac remodeling. Additionally, we sought to determine whether our model exhibits the expression of fundamental hypertrophic markers. Figure 4 shows that LMS incubated with TGF-β1 exhibit increased expression of ACTA2, the gene encoding alpha-smooth muscle actin (αSMA), a marker of myofibroblast differentiation. Preliminary data from immunofluorescence staining targeting αSMA support this finding at the protein level, suggesting a corresponding increase in αSMA-positive cells (Supplementary Fig. 3). In addition, we observed upregulation of TGFB1, as well as SMAD2 and SMAD3—key mediators of canonical TGF-β signaling—further indicating activation of a pro-fibrotic transcriptional program associated with fibroblast activation. Although none of the culturing conditions resulted in an upregulation of collagen types 1 and 3, (Supplementary Fig. 4), other genes related to ECM remodeling, such as fibronectin (*FN1*) and tenascin C (TNC), were upregulated in LMS cells cultured with TGF-β1. These changes could only be found in LMS cultivated with TGF-β1, regardless of their preload. However, only LMS subjected to combined stimulation with OL and TGF-β1 exhibited increased expression of the hypertrophic marker gene NPPB, which encodes B-type natriuretic peptide. This condition also led to upregulation of genes associated with mechanical stress, including Rho-associated protein kinase 2 (ROCK2) and focal adhesion kinase (FAK), indicating activation of mechanotransduction signaling pathways.

Overall, these findings indicate that LMS cultured under OL and TGF-β1 exhibit overexpression of key marker genes associated with cardiac fibrotic processes, such as fibroblast activation, cardiac remodeling, hypertrophy, and mechanical stress. This similarity of phenotype development compared to in vivo conditions underscores translational relevance of our ex vivo model for understanding of early cellular and molecular fibrotic changes, which could be potentially cured. Furthermore, this model provides a platform for accurate application of distinct mechanical and chemical factors for exploration of their role in the remodeling processes in myocardial tissue.

## Discussion

Investigation of heart failure processes in vitro, along with the associated hypertrophic and fibrotic changes, has been greatly hindered by the lack of experimental models that reflect in vivo conditions. While in vivo models are considered the benchmark for scientific research, they often obscure the specific contributions and interplay of individual factors due to their multifaceted nature. Here, we aimed to establish a novel ex vivo model to address this limitation, enabling precise control of mechanical and chemical stimuli, to investigate the effects of load-induced and receptor-mediated fibrosis in cardiac tissue. Although human LMS represent the most translational model, their broader application is currently limited by issues such as advanced remodeling in most available samples, restricted access to structurally preserved donor myocardium, and high inter-patient variability, underscoring the importance of large animal models as a translational bridge. We utilized LMS derived from porcine left-ventricular tissue combining the multicellular complexity and physiological characteristics of the heart in addition to the long-term viability and translational relevance of porcine heart tissue. To explore hypertrophic and fibrotic changes, we applied an overload (OL) to the LMS and added TGF-β1 to the culturing medium. The OL condition was achieved by culturing the tissue in BMCCs, which enabled continuous monitoring of the LMS contraction. This allowed for precise modulation of preload to reach a pathological level. Compared to similar approaches that use varying degrees of pre-stretch in LMS to achieve a specific sarcomere length^53,54^, our method enables preload adjustment tailored to the unique cellular and ECM composition of each LMS. Furthermore, our model offers controlled approach to accurately investigate factors contributing to cardiac fibrosis development, while avoiding pain, and suffering of animals during conventional procedures to induce cardiac remodeling processes^55^. Similar to the in vivo models of cardiac overload, our model also exhibited impaired contractile function, as shown by reduced contractility and a decreased maximum slope, both indicative of compromised systolic function. These findings not only align with those of Nunez et al., who reported contractile changes after mechanical overload in rat LMS^53^, but also with heart function in patients suffering from chronic cardiac overload^49,50^. The observed increase in the maximal slope of contraction in LMS cultivated under PL with TGF-β1 stimulation at 24 h and 72 h may be attributed to the electrophysiological effects of TGF-β1 on both myofibroblasts and cardiomyocytes. Previous studies have demonstrated that TGF-β1 not only modulates the crosstalk between these cell types but also influences cardiomyocyte ion channel expression. For instance, TGF-β1 released by myofibroblasts can alter sodium and potassium channel transcription and function, resulting in prolonged action potential duration and the emergence of early afterdepolarizations. Additionally, TGF-β1 affects the expression of connexins, thereby modifying gap junctional communication and intercellular electrical coupling. These combined effects on excitation–contraction coupling may contribute to the increased contractile slope observed in our LMS under pro-fibrotic stimulation^56–58^.

Furthermore, our model demonstrated structural and histological remodeling of the cardiac tissue when cultured under OL and TGF-β1 stimulation for 72 h. Namely, the increase of collagen deposition observed in our model serves as a classic hallmark of cardiac fibrosis and remodeling leading to heart failure^59^ and was accompanied by a significant enlargement of cardiomyocyte cross-sectional area, indicating early hypertrophic changes^60^. The elevated secretion of miR-21 in TGF-β1 stimulated LMS additionally serves as a key indicator of pro-fibrotic alterations within the tissue^51^, a finding previously observed by us in other LMS models upon acute or chronic injury^45,61^. Although it is known that TGF-β1 stimulation results in expression of miR-21 in cardiac fibroblasts and immune cells^62,63^, it has not been shown in a 3D model like LMS before and prompted us to investigate markers of fibroblast activation. Here, we identified the upregulation of several genes associated with early fibrotic changes and activation of fibroblasts, including *ACTA2*,* FN1*,* TNC*,* TGFB1*,* SMAD2 and SMAD3. ACTA2*, encoding alpha-smooth muscle actin, a critical marker of contractile fibroblasts, plays a pivotal role in fibroblast transformation to myofibroblasts and tissue remodeling^64^. *FN1* encoding fibronectin, an extracellular matrix protein facilitating cell adhesion and migration, supports fibrotic matrix deposition^22^. *TNC*, encoding tenascin C, modulating cellular behavior during tissue repair and fibrosis through its interaction with the extracellular matrix^23^. Lastly, *TGFB1* encoding TGF-β1 protein, a central regulator of fibroblast activation and collagen deposition via the two intracellular mediators of canonical TGF-β signaling *SMAD2* and *SMAD3*^30^. Together, the increased expression of these genes suggests enhanced fibroblast activation and migration, driving cardiac fibrosis and contributing to the maladaptive remodeling of cardiac tissue, as further supported by αSMA immunofluorescence staining in Supplementary Fig. 3. Moreover, increased expression of *NPPB*, encoding BNP^16^, along with *ROCK2* and *FAK*, was observed in mechanically stimulated LMS. *ROCK2* and *FAK* are key effectors of mechano-transduction signaling: *ROCK2* mediating cytoskeletal tension and contractility in response to mechanical cues, while *FAK* integrates signals from integrin-mediated adhesion sites to coordinate cellular responses to substrate stiffness and stress^65,66^. While the named pro-fibrotic genes were expressed only in the presence of TGF-β1 in the culture medium, regardless of the preload, *NPPB*,* ROCK2* and *FAK* was found to be significantly increased mostly in LMS that were subjected to additional stimulation with OL. TGF-β1 alone is a key regulator of the fibrotic microenvironment, where it not only activates fibroblasts but also induces endothelial to mesenchymal transition, whereby fibroblast-like cells lose their endothelial characteristics^67^, a process, which is also known to be regulated, in part, by miR-21^52^. These fibroblast-like cells could additionally contribute to the increase in expression of the mentioned pro-fibrotic genes, given the multicellular nature of the LMS and highlight further the role of TGF-β1. The fact that *NPPB*,* ROCK2 and FAK* expression was upregulated only when the tissue was exposed to both OL and TGF-β1 suggests that the hypertrophic response may be influenced by the synergistic effects of these stimuli, highlighting the complex interplay of factors driving fibrotic processes and heart failure in vivo. This further implicates the role of TGF-β1 in modulating the cellular phenotype in our model and highlights the value of complex 3D systems that preserve the native structural and physiological complexity of the myocardium.

While our study provides valuable insights into fibroblast activation and the molecular mechanisms underlying cardiac fibrosis and hypertrophy, there are certain limitations that warrant consideration. We acknowledge that our model system is inherently artificial and does not fully replicate the physiological conditions under which cardiac fibrosis and hypertrophy develop. In vivo, these processes typically unfold over months or even years, whereas our ex vivo stimulation approach accelerates this timeline considerably. Moreover, the LMS model does not capture the full transmural heterogeneity of intact myocardium, as slices are prepared from a specific wall region, and we did not investigate potential changes in tissue anisotropy that may also contribute to stiffness. Previous work has demonstrated that myocardial contractility is highly anisotropic and differs substantially between fiber, tissue, and organ levels, underscoring the importance of multiscale approaches to understand how local alterations translate into impaired systolic function in disease. While our study was not designed to address these directional aspects of contractility, incorporating such analyses in future work may provide additional mechanistic insight into stiffness development and contractile impairment in LMS^68^. Besides, stimuli such as mechanical stress and TGF-β1 signaling would normally occur in a temporally and spatially coordinated manner, rather than being applied simultaneously and at comparatively high concentrations, as in our experimental setup. Another limitation relates to the assessment of collagen expression. While PSR staining revealed increased collagen accumulation in TGF-β1-stimulated LMS, we were unable to detect a significant upregulation of collagen type I or III at the transcript or protein level (**Supplementary Figs. 4 and 5**). We hypothesize that this discrepancy may reflect a reduction in extracellular matrix (ECM) degradation rather than increased collagen synthesis. This is consistent with findings from other studies, which suggest that fibroblasts require a period of up to 7 days to reach the peak of collagen expression in mice hearts after myocardial infarction^19^. Consequently, the lack of detectable collagen changes on an RNA or protein level in our model could be related to the timing of the analysis. In vivo, cardiac remodeling and left ventricular stiffening can take years to develop, underscoring the limitation of our model in capturing rather early changes of phenotype development. However, this interpretation remains speculative, and future studies should incorporate more robust methods for ECM quantification, as Western blot analyses in our hands were inconclusive. Nevertheless, the sustained tissue integrity observed in our experiments, combined with our findings, suggests that the model represents early stages of fibrosis and hypertrophy, highlighting the need for long-term studies to fully elucidate ECM remodeling dynamics in response to fibroblast activation. Still, our findings reveal critical molecular pathways involved in cardiac fibrosis, as demonstrated by the upregulation of pro-fibrotic genes such as *ACTA2*,* FN1*, and *TNC*, in TGF-β1 stimulated LMS as well as the increase in expression of the hypertrophic marker gene *NPPB* under combined OL and TGF-β1 stimulation. Along with the impaired contractile performance and increased fibrotic matrix found in combination of OL and TGF-β1 stimulations, these results emphasize the interplay between mechanical and biochemical stimuli in driving fibrotic and hypertrophic remodeling processes, providing a foundation for further investigation.

Future studies could further explore the long-term effects of both here applied stimuli to better understand the dynamics of deterioration in the contractile function, extracellular matrix deposition and degradation, while also investigating additional hypertrophic characteristics. The use of porcine LMS offers a versatile and physiologically relevant model to unravel pathways related to cardiac fibrosis, hypertrophy, and heart failure. Beyond mechanistic insights, this model offers high potential for preclinical studies, such as drug testing, and the identification of novel therapeutic targets to mitigate pathological remodeling and to improve outcomes for patients with heart diseases.

## Supplementary Information

Below is the link to the electronic supplementary material.

Supplementary Material 1

## Data Availability

The datasets generated during and/or analyzed during the current study are available from the corresponding author on reasonable request.
