# Supplementary material for: Increase in mechanical load and pro-fibrotic stimulation leads to fibrotic and hypertrophic remodeling in porcine living myocardial slices
Source: Sci Rep. 2025 Nov 21;15:41180. doi: 10.1038/s41598-025-28222-z (PMC12639015; doi:10.1038/s41598-025-28222-z)
Supplement: Supplementary file 1 — Supplementary Material 1 [file 41598_2025_28222_MOESM1_ESM.pptx]

## Slide 1
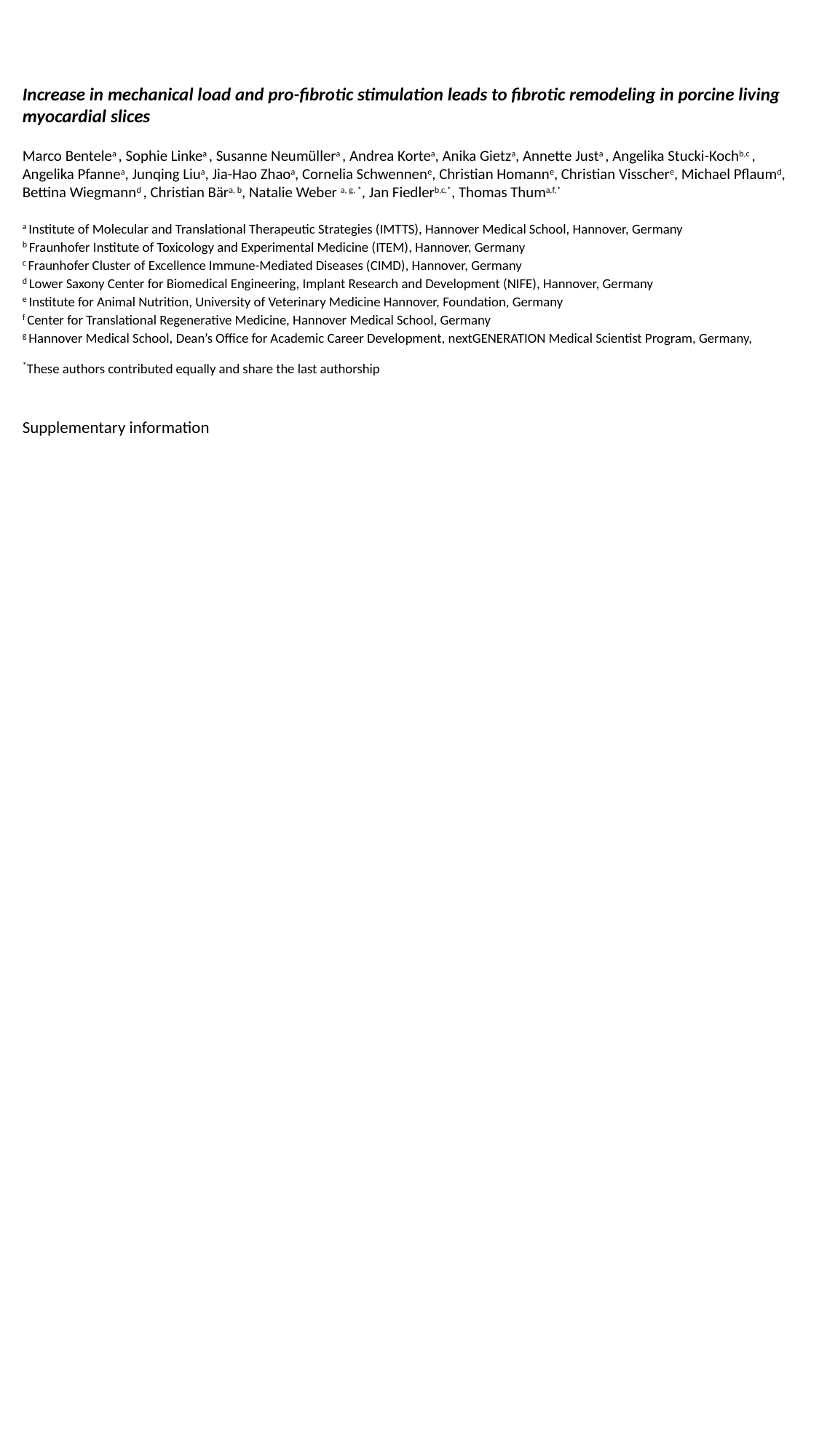

Increase in mechanical load and pro-fibrotic stimulation leads to fibrotic remodeling in porcine living myocardial slices
Marco Bentelea , Sophie Linkea , Susanne Neumüllera , Andrea Kortea, Anika Gietza, Annette Justa , Angelika Stucki-Kochb,c , Angelika Pfannea, Junqing Liua, Jia-Hao Zhaoa, Cornelia Schwennene, Christian Homanne, Christian Visschere, Michael Pflaumd, Bettina Wiegmannd , Christian Bära, b, Natalie Weber a, g, *, Jan Fiedlerb,c,*, Thomas Thuma,f,*
a Institute of Molecular and Translational Therapeutic Strategies (IMTTS), Hannover Medical School, Hannover, Germany
b Fraunhofer Institute of Toxicology and Experimental Medicine (ITEM), Hannover, Germany
c Fraunhofer Cluster of Excellence Immune-Mediated Diseases (CIMD), Hannover, Germany
d Lower Saxony Center for Biomedical Engineering, Implant Research and Development (NIFE), Hannover, Germany
e Institute for Animal Nutrition, University of Veterinary Medicine Hannover, Foundation, Germany
f Center for Translational Regenerative Medicine, Hannover Medical School, Germany
g Hannover Medical School, Dean’s Office for Academic Career Development, nextGENERATION Medical Scientist Program, Germany,
*These authors contributed equally and share the last authorship
Supplementary information

## Slide 2
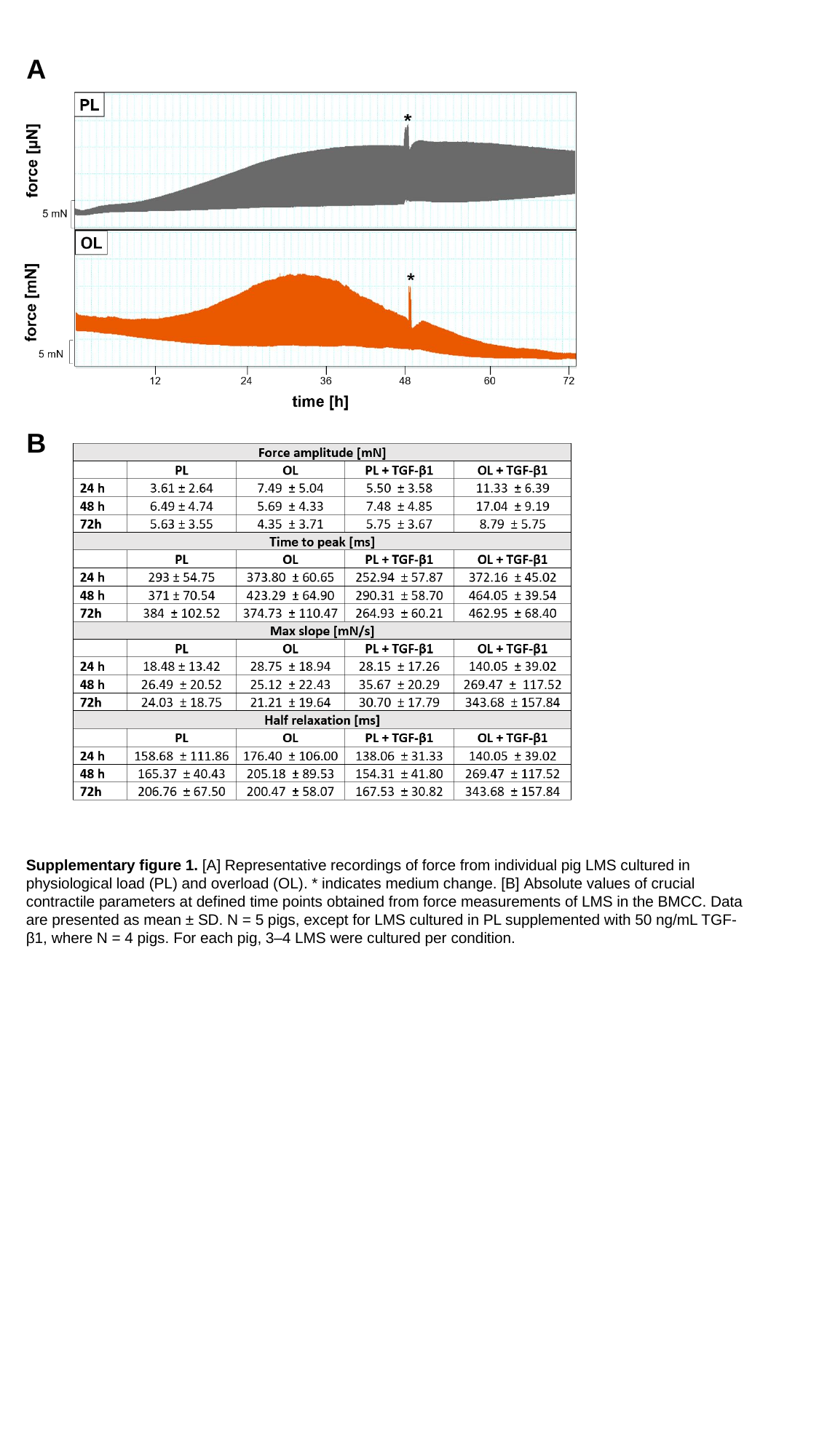

A
B
Supplementary figure 1. [A] Representative recordings of force from individual pig LMS cultured in physiological load (PL) and overload (OL). * indicates medium change. [B] Absolute values of crucial contractile parameters at defined time points obtained from force measurements of LMS in the BMCC. Data are presented as mean ± SD. N = 5 pigs, except for LMS cultured in PL supplemented with 50 ng/mL TGF-β1, where N = 4 pigs. For each pig, 3–4 LMS were cultured per condition.

## Slide 3
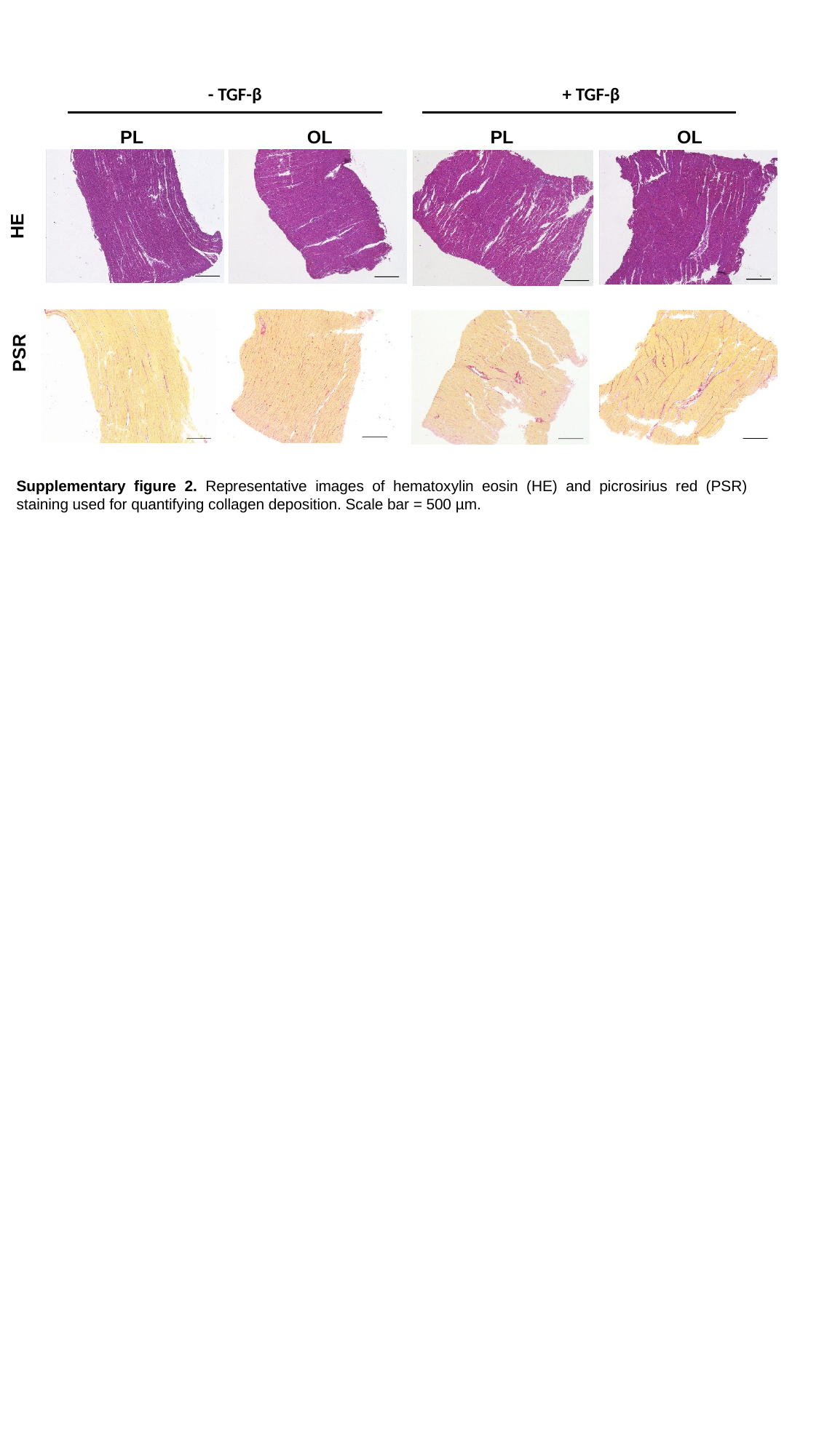

- TGF-β
+ TGF-β
PL
OL
PL
OL
HE
PSR
Supplementary figure 2. Representative images of hematoxylin eosin (HE) and picrosirius red (PSR) staining used for quantifying collagen deposition. Scale bar = 500 µm.

## Slide 4
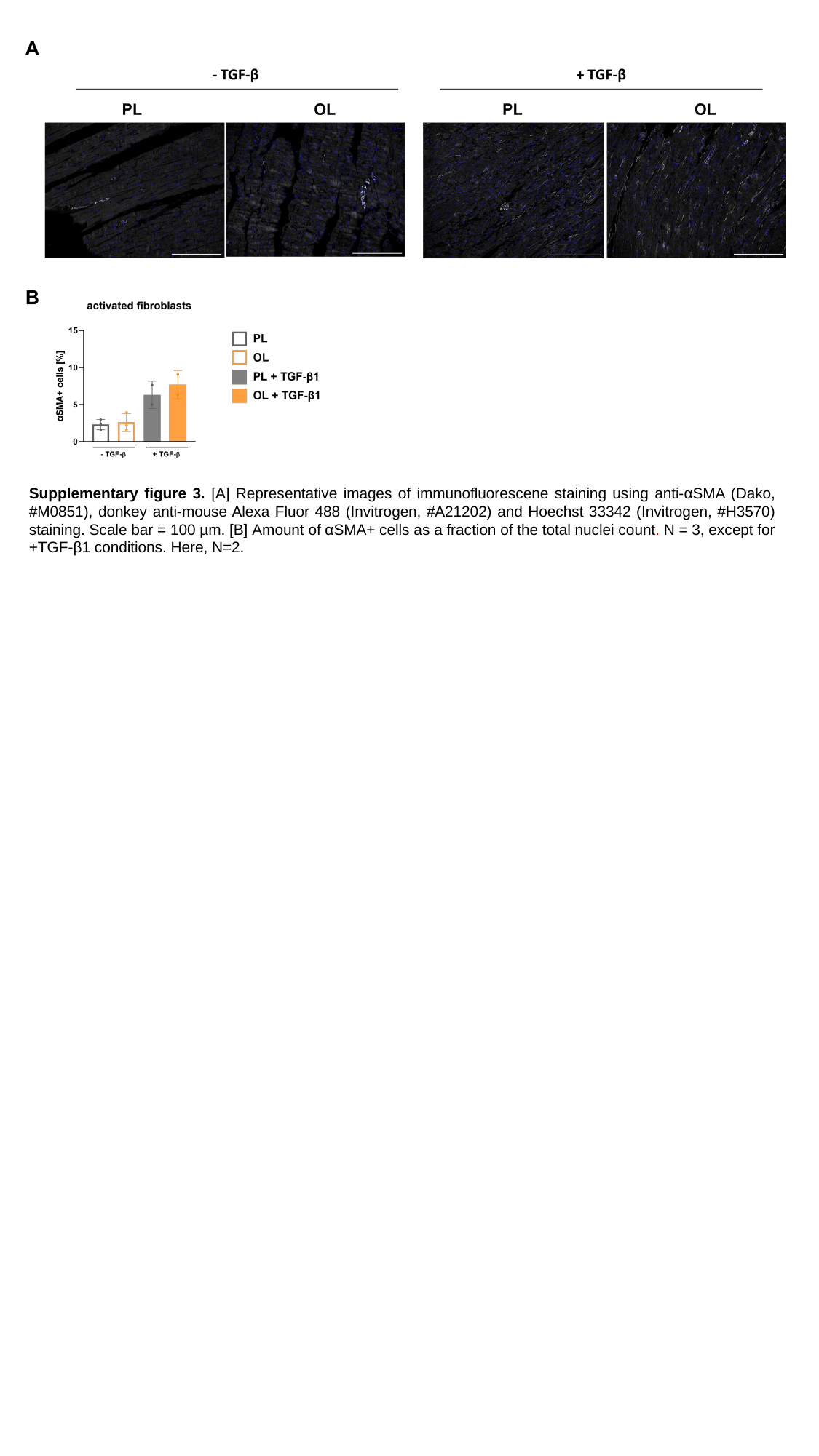

Supplementary figure 3. [A] Representative images of immunofluorescene staining using anti-αSMA (Dako, #M0851), donkey anti-mouse Alexa Fluor 488 (Invitrogen, #A21202) and Hoechst 33342 (Invitrogen, #H3570) staining. Scale bar = 100 µm. [B] Amount of αSMA+ cells as a fraction of the total nuclei count. N = 3, except for +TGF-β1 conditions. Here, N=2.

## Slide 5
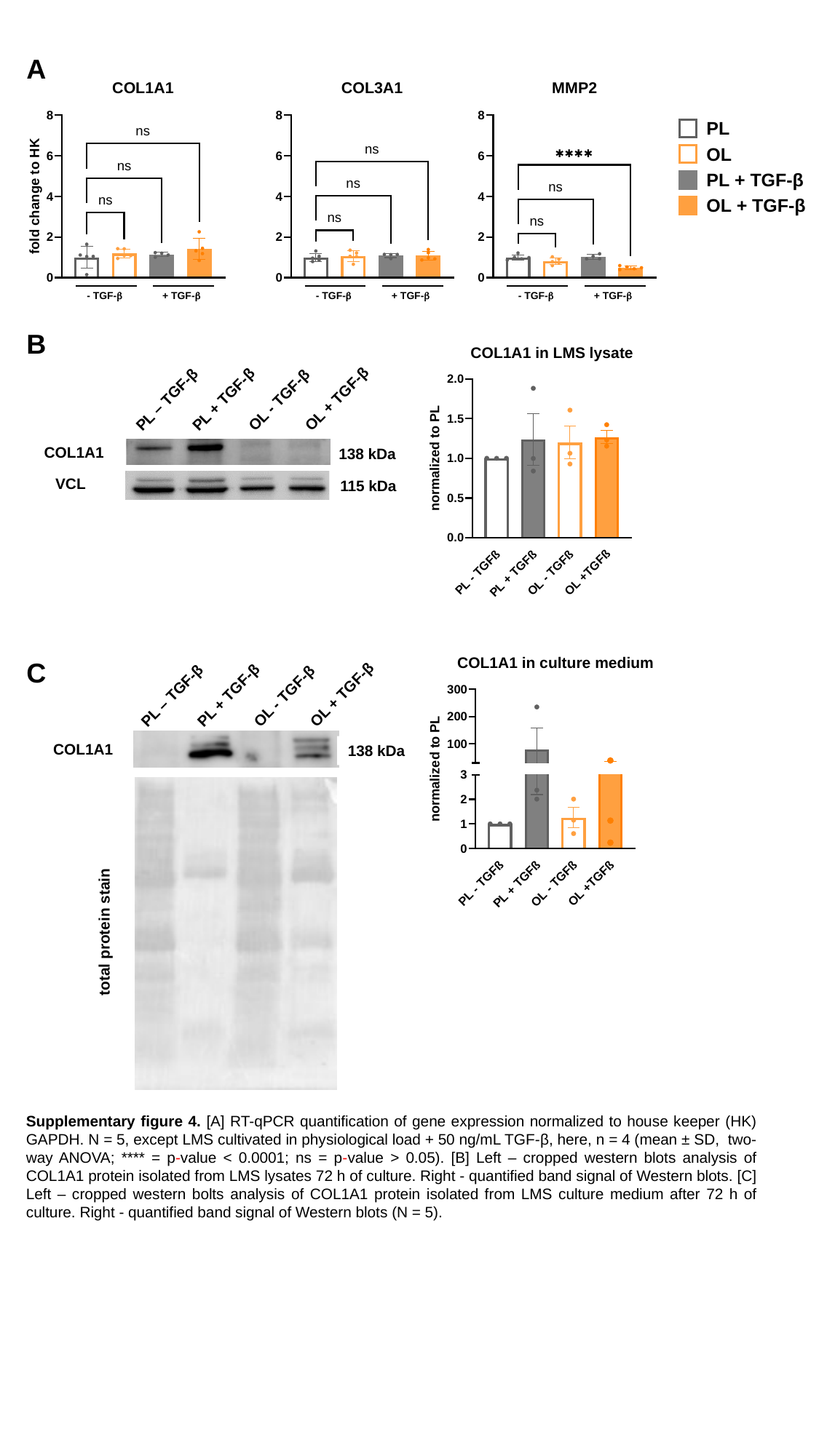

A
PL – TGF-β
PL + TGF-β
OL + TGF-β
OL - TGF-β
COL1A1
138 kDa
VCL
115 kDa
B
PL – TGF-β
PL + TGF-β
OL + TGF-β
OL - TGF-β
COL1A1
138 kDa
total protein stain
C
Supplementary figure 4. [A] RT-qPCR quantification of gene expression normalized to house keeper (HK) GAPDH. N = 5, except LMS cultivated in physiological load + 50 ng/mL TGF-β, here, n = 4 (mean ± SD, two-way ANOVA; **** = p-value < 0.0001; ns = p-value > 0.05). [B] Left – cropped western blots analysis of COL1A1 protein isolated from LMS lysates 72 h of culture. Right - quantified band signal of Western blots. [C] Left – cropped western bolts analysis of COL1A1 protein isolated from LMS culture medium after 72 h of culture. Right - quantified band signal of Western blots (N = 5).

## Slide 6
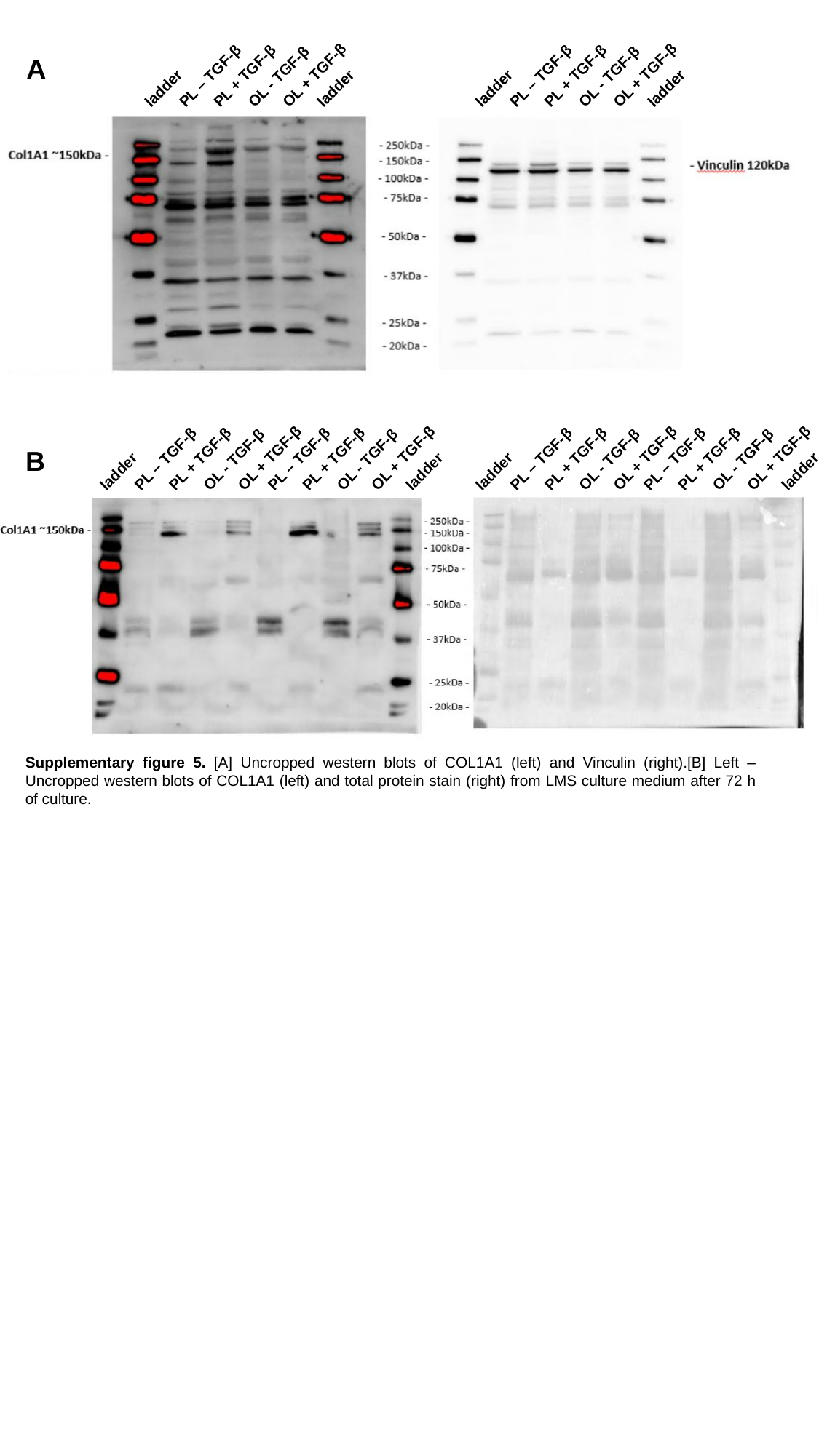

A
ladder
PL – TGF-β
PL + TGF-β
OL + TGF-β
ladder
ladder
PL – TGF-β
PL + TGF-β
OL + TGF-β
ladder
OL - TGF-β
OL - TGF-β
ladder
PL – TGF-β
PL + TGF-β
OL - TGF-β
OL + TGF-β
PL – TGF-β
PL + TGF-β
OL - TGF-β
OL + TGF-β
ladder
ladder
PL – TGF-β
PL + TGF-β
OL - TGF-β
OL + TGF-β
PL – TGF-β
PL + TGF-β
OL - TGF-β
OL + TGF-β
ladder
B
Supplementary figure 5. [A] Uncropped western blots of COL1A1 (left) and Vinculin (right).[B] Left – Uncropped western blots of COL1A1 (left) and total protein stain (right) from LMS culture medium after 72 h of culture.
